# Comparative genomics for biodiversity conservation

**DOI:** 10.1016/j.csbj.2015.05.003

**Published:** 2015-05-21

**Authors:** Catherine E. Grueber

**Affiliations:** Faculty of Veterinary Science, University of Sydney, NSW, Australia; San Diego Zoo Global, San Diego, CA, USA

**Keywords:** Adaptation, Disease, Hybridisation, Management units, Phylogeny, Threatened species

## Abstract

Genomic approaches are gathering momentum in biology and emerging opportunities lie in the creative use of comparative molecular methods for revealing the processes that influence diversity of wildlife. However, few comparative genomic studies are performed with explicit and specific objectives to aid conservation of wild populations. Here I provide a brief overview of comparative genomic approaches that offer specific benefits to biodiversity conservation. Because conservation examples are few, I draw on research from other areas to demonstrate how comparing genomic data across taxa may be used to inform the characterisation of conservation units and studies of hybridisation, as well as studies that provide conservation outcomes from a better understanding of the drivers of divergence. A comparative approach can also provide valuable insight into the threatening processes that impact rare species, such as emerging diseases and their management in conservation. In addition to these opportunities, I note areas where additional research is warranted. Overall, comparing and contrasting the genomic composition of threatened and other species provide several useful tools for helping to preserve the molecular biodiversity of the global ecosystem.

## 1 Introduction

Conservation genetics has entered the world of genomics [1]. The number of species with whole-genome sequence data is continually growing [2,3], so that more and more endangered taxa are becoming “genome-enabled” [4], that is, genome resources are available for them or their close relatives. These new technologies provide researchers with unprecedented levels of data to generate precise estimates of essential population genetic parameters, to examine questions such as the causes and genetic consequences of population decline and fragmentation [1,5; for critique see 6]. Most applied conservation genetics research targets issues operating within or amongst populations of the same species (which may be spatially or temporally separated) [7]. This level of focus is often appropriate because anthropogenic threatening processes typically occur over relatively short evolutionary time frames: the scale relevant to population/species-level processes rather than deeper evolutionary trajectories such as speciation. Nevertheless, there is additional insight to be gained from considering the evolutionary context of threatened species, i.e. by taking a comparative approach across taxa. For example, comparative analyses of species' demographic and life history characteristics have revealed those particular ecological traits that predispose species to high risk of extinction [8,9]. In this paper, I explore how comparative approaches using genomic data may also add value to conservation efforts.

Comparative genomics benefits most from high-quality, annotated and mapped genome data, but the pre-existence of such complete data is not necessarily a prerequisite for taking a comparative genomic approach to wildlife genetics [10]. This is good news for conservation scientists, who frequently work on non-model species for which genome resources do not exist. Several options are available, although not all will be uniformly applicable across contexts: reduced-representation libraries [11] provide a cost-effective means of obtaining genome-level data for comparative studies [12]. RNAseq to obtain transcriptome-level data can also provide valuable insight, without necessarily obtaining whole-genome data (e.g. comparative RNA sequencing of 12 primate species, most of which had little or no genomic resources [13]). Genome-informed SNP arrays, developed for well-studied species, can be used to generate large amounts of data for closely related threatened taxa (e.g. utilising a primarily domestic dog SNP array to study wild canids [14]), although the percentage of shared polymorphisms between species decays exponentially with divergence time, decreasing the amount of data obtained from the chip for more distant species [15]. A further approach to preliminary comparative genomics investigation is the generation of large amounts of sequencing data, which is then aligned to the annotated genome of a closely related species (e.g. aligning California condor sequencing data against the chicken genome [16]).

Wildlife genomics may be undertaken at multiple levels, from comparing individuals within a population (in a population genetics/genomics framework) to comparisons at higher taxonomic levels (comparative genomics). Many genomics techniques offer opportunities for conservation (for recent overviews, see [1,3,6,17]). However, despite their potential value, comparative genomic studies with explicit and specific conservation applications remain uncommon ([1,6,18], exceptions are [19,20]). Impediments to the uptake of genomics in conservation include sampling and analysis constraints [21], as well as a lack of clear examples of successful application [6], amongst others. In this review, I focus on possible applications of comparative genomics to conservation, and provide examples of a variety of avenues for future work in this field. Comparative genomics itself is a broad field, with the potential to answer many salient questions in evolutionary biology, medicine, and other fields (e.g. [22]), and therefore the analyses mentioned herein also have many applications beyond threatened species management. In fact, due to the scarcity of conservation examples, much of the empirical work I discuss here has been conducted on non-threatened species. I touch on a number of topics in brief: my aim is not to provide an exhaustive survey, but rather an overview of new ways that an ever-growing resource of genomic data can be exploited to address timely problems in biodiversity conservation.

### 1.1 Applications of comparative genomics to conservation

My main discussion centres on a summary of conservation science research questions that may be approached or supported by the use of comparative genomic methods, and identification of research needs to further progress these aims.

#### 1.1.1 Characterisation of conservation units

Identifying units of conservation is a fundamental goal of any conservation strategy, essential to both resource planning in a legal and financial sense (e.g. how to distribute conservation effort) and management planning in a practical and biological sense (e.g. which populations may be mixed and which show important distinctiveness that should be preserved). Although definitions vary [23], the concept of conservation management units encompasses groupings beyond traditional taxonomic demarcations, such as evolutionarily significant units and/or variants with particular ecological or social value. Nevertheless, conservation units are usually informed by phylogeny, traditionally using putatively neutral genetic regions such as microsatellite markers or mtDNA. Importantly, these methods inform conservationists as to the degree of migration amongst putative conservation units [24] providing a distinction between “evolutionarily significant units”: populations that are phylogenetically discrete, and “management units”: populations with significant divergence in allele frequencies [25].

Recently, researchers have begun to target adaptive molecular variation for inclusion in the assessment of conservation units. These data introduce information about evolutionary distinctiveness into the definition of protected populations [26]. For example, diversity and differentiation at the major-histocompatibility complex (MHC), genes associated with adaptive immunity [27], have been incorporated into the delineation of conservation management units for several species, such as giant panda *Ailuropoda melanoleuca* [28] and marbled murrelets *Brachyramphus marmoratus* (a threatened seabird) [29]. However, basing management decisions on a small number of functional genomic regions presents a high risk of failing to detect evolutionarily and ecologically important processes that influence other parts of the genome [30]. Recent studies have shown how genome-level data can provide very high resolution for the reconstruction of phylogenetic trees, enabling detailed identification of species boundaries and relationships [12,31]. For example, Wagner et al. [31] recently used reduced-representation RAD sequencing to generate exceptionally detailed phylogenetic inference amongst 16 cichlid species in Lake Victoria, a community well-studied in evolutionary ecology. Evolutionary relationships amongst these species had previously been difficult to dissect using traditional methods, due to very recent divergence times which impaired discrimination amongst morphologically distinct species using much smaller numbers of nuclear and mitochondrial DNA sequence variants [31].

In conservation, phylogenetic approaches have been used to identify the most evolutionarily distinct species, which may then be targeted for particular conservation effort (e.g. EDGE [evolutionarily distinct, globally endangered] species [32]). Taking a whole-genome comparative approach to the characterisation of conservation units provides at least three advantages over traditional approaches: 1) greater resolution via the use of many more loci, 2) the ability to incorporate a wide diversity of putatively functional genetic regions (i.e. genic sequences) and 3) the ability to perform analyses using either neutral or functional data (or both), enabling researchers to study how different processes drive population structure [33]. Several challenges exist with the use of whole-genome data for the reconstruction of phylogenetic trees, such as how to conduct inference regarding species trees in the case of conflicting gene trees from different genomic regions [34]. These issues apply to all studies that use multigene data for phylogenetics, not just those with conservation aims, and their resolution is still an area of active research (e.g. [34,35]). Nevertheless, genome-level data enables researchers to determine whether any differentiation observed amongst populations results from evolutionary or demographic processes. For example, genetic structures based on different genomic regions (such as microsatellites versus MHC) are frequently uncorrelated (e.g. [29]), typically interpreted as a greater role of selection than drift at immunogenetic versus neutral loci, respectively [36]. Populations differing as a result of recent, drift-associated processes are not considered as distinct as populations differing as a result of deep adaptation processes [23]. Differentiating these mechanisms of structure amongst populations is essential to the fully informed preservation and management of molecular biodiversity in ecosystems [1].

#### 1.1.2 Informing the conservation consequences of hybridisation

Human landscape modification has increased the frequency with which hybridisation influences the evolutionary course of many species around the world [37]. Introgression of a threatened species by a previously geographically separated and more-common relative can affect species integrity and result in extinction of the rarer type [38,39]. Although the term “hybridisation” typically refers to interspecific breeding, it has also been used to refer to interbreeding at lower levels of genetic differentiation, which may be of conservation relevance, such as amongst sub-species or regional variants. Hybridisation between previously separated groups may not only result in the extinction of rare forms by assimilation, but also accelerate the decline of threatened populations via outbreeding depression: the decreased fitness that occurs when distantly related lineages interbreed, as a result of the break-up of locally adapted haplotypes or gene combinations [40–42]. Determining the evolutionary timing of hybridisation is important from a conservation perspective, in order to evaluate whether it has occurred as a result of natural or anthropogenic processes [39]. Population and comparative genetics can be used to quantify the degree of ancient or recent hybridisation between threatened and non-threatened species [14,43], as well as to identify taxon-specific markers for the diagnosis and monitoring of hybridisation in dynamic systems (e.g. [44]). Identifying the degree of hybridisation represented by particular individuals, and thus whether any “pure forms” remain in a population, is essential in a conservation context for determining whether pure lineages can be recovered [39].

Studying hybridisation in some taxonomic groups requires molecular markers at the genome-level due to peculiar characteristics of the species' genomic architecture (such as a recent [~ 25–100 MYA] genome duplication in salmonids [20], or polyploidization in plants [45]). In addition, research at the comparative genomic level can provide additional information that cannot be obtained using conventional genetic approaches: by examining extensive functional diversity data, researchers can identify the particular genomic regions that determine the physiological consequences of hybridisation. For example, diagnosing particular chromosomal regions under strong selection during hybridisation events allowed researchers to quantify the proportion of the wheat genome that has been affected by introgression [45]. Such studies can also inform whether particular genes are experiencing strong selection as a result (e.g. the identification of “super invasive” alleles at higher-than-expected frequencies in admixed populations of salmonids [46]).

Identifying genomic regions most affected by introgression may also provide diagnostic markers for rapidly distinguishing hybrids from pure forms [46]. These approaches have been used to inform the management of westslope cutthroat trout *Oncorhynchus clarki lewisi* in North America [20,46], for which the major threat to persistence is hybridisation with introduced rainbow trout *O. mykiss* [47]. Researchers used genome-level data to identify 3180 species-diagnostic SNPs, which were then genotyped in fish from multiple populations to evaluate levels of introgression and numbers of pure forms remaining [20,46]. The data also provided estimates of the proportion of the genomes of individual hybrid fish that could be traced back to cutthroat or rainbow trout. Quantifying individual-level introgression in this way enabled the researchers to study introgression with great precision, including identification of those animals for which a very small proportion of their genome came from the invasive species, as well as the discovery of candidate adaptive regions experiencing strong selection pressure during the hybridisation process [46].

Overall, the comparative genomic approach can provide unprecedented levels of precision for the study of hybridisation in conservation contexts, providing diagnostic markers for the identification of hybrids versus pure forms. Comparative genome-level analyses have the potential to reveal the genomic architecture of hybridisation, for the purpose of understanding the evolutionary mechanisms that drive hybrid genome evolution. From a practical perspective, these findings can also predict the effectiveness of novel selective breeding approaches to producing pure forms from recently introgressed populations as well as identifying those individuals to target (e.g. [48,49]). Molecular selective breeding may enable the recovery of genetic diversity contained within hybrids, which would otherwise be lost if only pure forms were targeted for breeding [49]. Using molecular genetics for the latter is preferred over pedigree or morphological approaches to preserving rare forms from hybridisation, which can result in very high levels of inbreeding [50], although there are advantages to integrating multiple data types, especially when there may be ascertainment bias in the reference genomic material relative to the target species (e.g. [51]). The use of genome-level molecular data for de-introgression is still in its infancy: so far it has been pursued theoretically using real molecular data from intentional hybrids of Merino and Poll Dorset sheep breeds [49], and proposed in conservation planning for the endangered Cika cattle breed, which has been historically hybridised with other breeds [51]. Computational modelling has shown that selective breeding regimes could ultimately recover pure genomes from introgressed populations in only a few generations [49]. It will be interesting to observe whether this approach, currently targeting the conservation of rare breeds of commercially important domestic species, will offer benefits for the recovery of wildlife species threatened by hybridisation in the future.

### 1.2 Discovering the drivers of divergence

A broad goal of comparative genomics is to reveal the evolutionary forces that drive genetic and genomic diversity and, as already shown, these findings can add value to specific conservation problems. Importantly, comparative approaches can reveal the genomic regions under selection and putative genetic underpinnings of unique traits [3,13,52]. Identifying regions of the genome that are more divergent than expected, as well as those regions that are more variable than expected, may help guide the preservation of genetic diversity in conservation management, especially in captivity. At present, little is known about the ongoing genetic consequences of adaptation to captivity in threatened species, particularly when captive-raised animals are reintroduced to the natural environment [23]. Comparing genomes amongst species can help to identify those regions that are experiencing particularly strong positive selection, which may signify rapid adaptation to captivity, providing markers for the management of this undesired process [23,53].

At the whole-genome level, comparative approaches can reveal gene losses/gains and help researchers discover the defining characteristics of species (e.g. [54,55]) as these changes are major contributors to functional evolution and divergence [56]. However, inference based on gene losses/gains requires very high-quality genomes [57]. Many conservation genomic studies use data obtained through reduced-representation methods or other unassembled data types [58], which would not be suitable for addressing these questions. It has also been noted that such “genome scan” population genomic studies need to be undertaken with caution, as the risk of false-positives is high [59]. Continued advances in the computational comparison of genomes, in order to more accurately catalogue regions of divergence between species (whether sequence divergence or gene gains/losses) may provide valuable information about the biology and life history of species (e.g. [54]). Identifying the genetic origin of unique traits can be helpful for managing threatening processes (e.g. determining whether they have intrinsic or extrinsic causes). For example, in comparing the giant panda genome to those of other carnivores, the absence of digestive cellulose enzymes enabled Li et al. [55] to infer that the strict bamboo diet of the species is unlikely to have arisen as a result of intrinsic genetic characteristics, and may be directed more by gut microbiota. In addition, identifying regions of shared synteny between related species can aid in the characterisation of highly divergent orthologous genes, which would be difficult to identify using homology searches alone [54]. Clearly, there are advantages to be gained from taking a comparative approach to discovering the most divergent loci, and a better understanding threatened species and their potential response to threats. Ongoing improvements to genomic assembly and comparison, particularly the identification of variation in gene composition amongst species, will help to facilitate this conservation research.

### 1.3 Understanding intrinsic threats and disease resilience

Over time, small, isolated populations will gradually lose genetic diversity and levels of inbreeding will increase: together, these processes erode the resilience of these populations to environmental changes and lead to increased expression of deleterious recessive alleles [60,61]. These genetic impacts leave populations vulnerable to a variety of novel threats, particularly diseases. Comparative genomic approaches can assist in the study of the disease threats that arise from losses in genetic diversity, by providing a greater understanding of the origin of diseases and the response of populations. The results can then be used to design strategies for disease treatment or management. In this section, I provide examples of research into diseases of threatened species that have applied comparative molecular approaches.

Comparative genomics of wildlife can support the identification of candidate loci responsible for heritable disorders that may increase in frequency in small populations as a result of inbreeding. An example is the lethal chondrodystrophy seen in the critically endangered California condor *Gymnogyps californianus* [16]. This disease showed Mendelian segregation in condor pedigrees [62], so researchers developed genomic resources with the aim of identifying the genetic basis of the disorder and revealing genetic markers linked to the disease [16,63]. It is hoped that this ongoing research will ultimately inform the design of captive breeding protocols that can reduce the frequency of chondrodystropy, although it would be important to avoid losses of genetic diversity overall (i.e. at other loci), and ensure inbreeding is minimised to avoid further decreases in individual fitness [23].

A particularly interesting application of genomics to the study of disease emergence in a threatened species is in the Tasmanian devil *Sarcophilus harrisi*, which is under threat from devil facial tumour disease (DFTD). DFTD is a highly contagious and fatal transmissible cancer that has caused dramatic population declines in the species' native Tasmania, since its first observation in 1996 [64]. Uniquely, DFTD cells are the infectious agent of the disease, spread via direct contact between individuals [65], probably when devils bite one another during aggressive interactions [66]. In this case, comparative genomic research compares the host genome with that of its “pathogen”, DFTD, via whole-genome sequencing [67]. The aims of this work are to identify the underlying mutations driving tumorigenesis and the ability of DFTD to evade the immune system of the host, with a long-term view toward a potentially identifying a target for a vaccine or other therapeutic [67,68]. Many insights into the origin, transmission and evolution of DFTD have arisen from this and related work, which is still ongoing [68]. Meanwhile, comparisons between the genome of DFTD and its host have enabled the identification of marker loci, which, through genotyping of large numbers of tumour samples from throughout the population, have confirmed the clonal origin of DFTD: it only arose once and has spread [67]. This result has had important conservation implications via the realisation that healthy devils could be prevented from acquiring the disease if they were physically separated from diseased animals – leading to the establishment of a highly successful captive “insurance” population [69].

In addition to these examples of particular disease processes, comparative genomics can be a powerful tool for the study of a species' immunome. By examining the diverse characteristics of species' immunity genes, we can learn more about how each species has evolved to respond to pathogens in its environment [70]. In a conservation context, the comparative approach can be used to address questions about the particular characteristics that predispose populations to emerging threats (as has been done from an ecological perspective [9]). For example, recent world-wide amphibian declines have resulted from the spread of the chytrid fungus *Batrachochytrium dendrobatidis* [71]. Comparative immunogenomics of *Xenopus* and closely related species allows researchers to examine the response of the model amphibians to chytrid and other pathogens, in order to discover how diseases may affect threatened species [72]. As whole-genome sequences are published for a greater number of species, the hope is that whole-immunome comparisons across species will become more refined, improving the power of studies examining the immunogenetic diversity, and therefore the disease consequences of low diversity, in threatened taxa [18]. At present, immune-gene regions are challenging to assemble, in part due to the very traits that make immunity highly adaptable: high levels of gene duplication [73,74]. It is therefore difficult to determine how many copies of duplicates genes are present in a species or even individual genome [75,76]. Emerging technologies offering longer sequencing reads [e.g. 77], as well as continued development and assessment of computational approaches [e.g. 78,79], may help to overcome this challenge.

## 2 Conclusions

I have presented a brief overview of conservation genetics questions that can be targeted by a comparative genomic approach: including the relationships between related species or populations (delineation of conservation units and studies of hybridisation), studies of cross-species variation (examination of the genomic regions contributing to species distinctiveness and divergence) and the interactions between species and their threatening processes (whether intrinsic or extrinsic). It is clear that many aspects of preserving threatened species diversity can benefit from looking beyond the threatened species itself, and considering the differences between the rare species' genome and other genomes that share its environment, although empirical examples in this research area, utilising threatened species themselves, are scarce. A particularly exciting path of inquiry is the application of comparative genomics to the study of the ecological context of genomes, through a better understanding of interactions between species, such as hosts and parasites, in an “extended phenotype”, co-evolutionary framework [80,81] (for examples see [82–84]). Such findings present promising opportunities to better understand the evolutionary consequences of anthropogenic biotic disturbances to ecosystems, such as the shifting distributions of pathogens, predators, prey and competitors. A better understanding of these complex ecological interactions can help us to understand the relationships amongst species and inform conservation planning at higher levels, such as the protection of vulnerable habitats and ecological processes. The preservation of biodiversity is essential at multiple levels: from molecular diversity, to species, to whole ecosystems and habitats [e.g. 85]. One of the best arguments for the use of comparative genomics in conservation is that whole-genome level data is necessary to monitor and protect the greatest breadth of genetic biodiversity [1]. By comparing and contrasting the processes that influence the genomic composition of threatened species with those of more-common species, we can identify what makes vulnerable species ecologically and genetically unique, and stand the best chance at preserving them and their individual roles in the global ecosystem.
